# Software Analysis of Colonoscopy Videos Enhances Teaching and Quality Metrics

**DOI:** 10.7759/cureus.23039

**Published:** 2022-03-10

**Authors:** Vasant Rajan, Havish Srinath, Christopher Yii Siang Bong, Alex Cichowski, Christopher J Young, Peter J Hewett

**Affiliations:** 1 Department of General Surgery, Logan Hospital, Meadowbrook, AUS; 2 Australian Institute for Machine Learning, The University of Adelaide, Adelaide, AUS; 3 Department of General and Colorectal Surgery, Memorial Health System, Abilene, USA; 4 Colorectal Surgery, Department of Surgery, The Queen Elizabeth Hospital, Adelaide, AUS

**Keywords:** pilot project, cohort studies, australia, software, colonoscopy

## Abstract

Purpose

Machine learning algorithms were hypothesized as being able to predict the quality of colonoscopy luminal images. This is to enhance training and quality indicators in endoscopy.

Methods

A separate study involving a randomized controlled trial of capped vs. un-capped colonoscopies provided the colonoscopy videos for this study. Videos were analyzed with an algorithm devised by the Australian Institute for Machine Learning. The image analysis validated focus measure, steerable filters-based metrics (SFIL), was used to assess luminal visualization quality and was compared with two independent clinician assessments (C1 and C2). Goodman and Kruskal's gamma (G) measure was used to assess rank correlation data using IBM SPSS Statistics for Windows, version 25.0 (IBM Corp., Armonk, NY).

Results

A total of 500 random colonoscopy video clips were extracted and analyzed, 88 being excluded. SFIL scores matched with C1 in 45% and C2 in 42% of cases, respectively. There was a significant correlation between SFIL and C1 (G = 0.644, p < 0.005) and SFIL and C2 (G = 0.734, p < 0.005).

Conclusion

This study demonstrates that machine learning algorithms can recognize the quality of luminal visualization during colonoscopy. We intend to apply this in the future to enhance colonoscopy training and as a metric for quality assessment.

## Introduction

Interest in quality within medicine and its subsequent use to improve healthcare delivery has grown markedly since the Institute of Medicine’s report on medical errors published in 2000 [1]. Colonoscopy is an excellent area for quality improvement given it is high volume with associated high risks and costs [2]. This has been recognized by the Australian Commission on Safety and Quality in Health Care, via the creation of the Colonoscopy Clinical Care Standard, which aimed to improve the safety and quality of colonoscopy via the identification and optimization of nine domains [3].

Other research in this area includes machine learning algorithms, which are theorized to minimize inter-observer variability in detecting and diagnosing luminal lesions [4]. Devices such as cap-assisted endoscopes are proposed to improve adenoma detection rate (ADR) by improving colonic mucosal visualization [5]. Artificial intelligence (AI) has been suggested as a method to improve ADR by capitalizing on deep learning, whereby AI is trained through repetitively studying recorded videos to recognize polyps that may occur only fleetingly during colonoscopies [6]. Such AIs are deployed to assist the endoscopist in detecting adenomas. Preliminary studies of these methods have shown dramatic improvements in ADR, from 40% in unassisted colonoscopies to 80% when an AI is deployed to assist in detection [7]. Oh et al. [8] have also proposed software analysis of colonoscopy video quality, which ultimately aims to provide a new standard against which the quality of colonoscopies can be objectively scored. This would assist in quality control measures on a large scale and eventually serve as a baseline against which benchmark can progress in colonoscopy training.

Our aim was to use machine learning algorithm software to predict luminal clarity quality while minimizing inter-observer variability. In theory, this would lead to greater standardization and objectivity in assessing colonic luminal visualization.

## Materials and methods

A previously conducted randomized controlled trial at the Queen Elizabeth Hospital, Adelaide, South Australia, designed to assess differences in ADRs between uncapped and cap-assisted endoscopes, provided the colonoscopy video content for this study. As part of the trial, the procedures were recorded. Ethics approval was obtained from the local Human Research Ethics Committee. The colonoscopes used in the study were the Olympus CF-H190L/I and CF-H180L/1 adult scope (Olympus Medical Systems, Tokyo, Japan), on which were attached the ARV120 Endocuff Vision, and the PCF-H180AL/I and PCF-H190L/I, which were attached with the ARV180 Endocuff Vision [5]. A total of 214 videos were obtained from the process and 32 videos were excluded for reasons that included obstructing cancers, strictures, consent, and device issues.

An algorithm was developed to design a software product that can be objective and have global applicability across different platforms and devices. We envisaged a software program that will require minimal input from the user and can be utilized with ease by clinicians of varying experience. Video analysis was performed in collaboration with the Australian Institute for Machine Learning, based at the University of Adelaide. Preliminary research assessed the optimum way of grading image focus.

**Figure 1:**
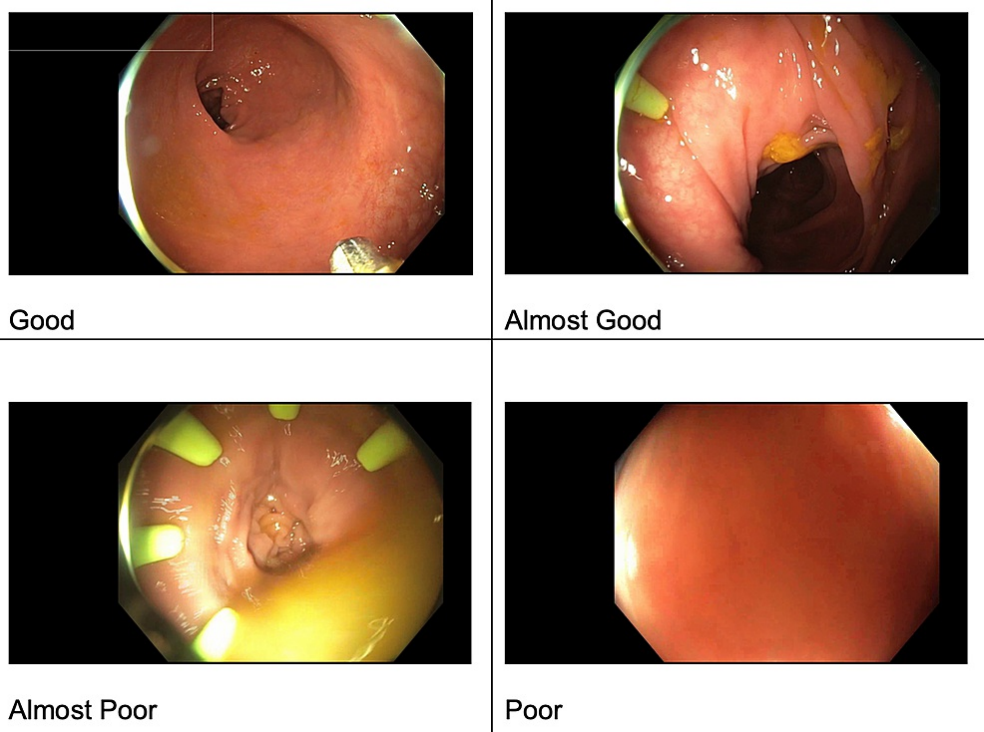
Examples representative of image quality noted during colonoscopy curated by experienced endoscopists.

The first steps in video selection included curating a set of images from experienced endoscopists that were representative of image quality noted during colonoscopy. The endoscopists classified the images into a four-point Likert scale: good, almost good, almost poor, and poor (Figure 1).

**Figure 2:**
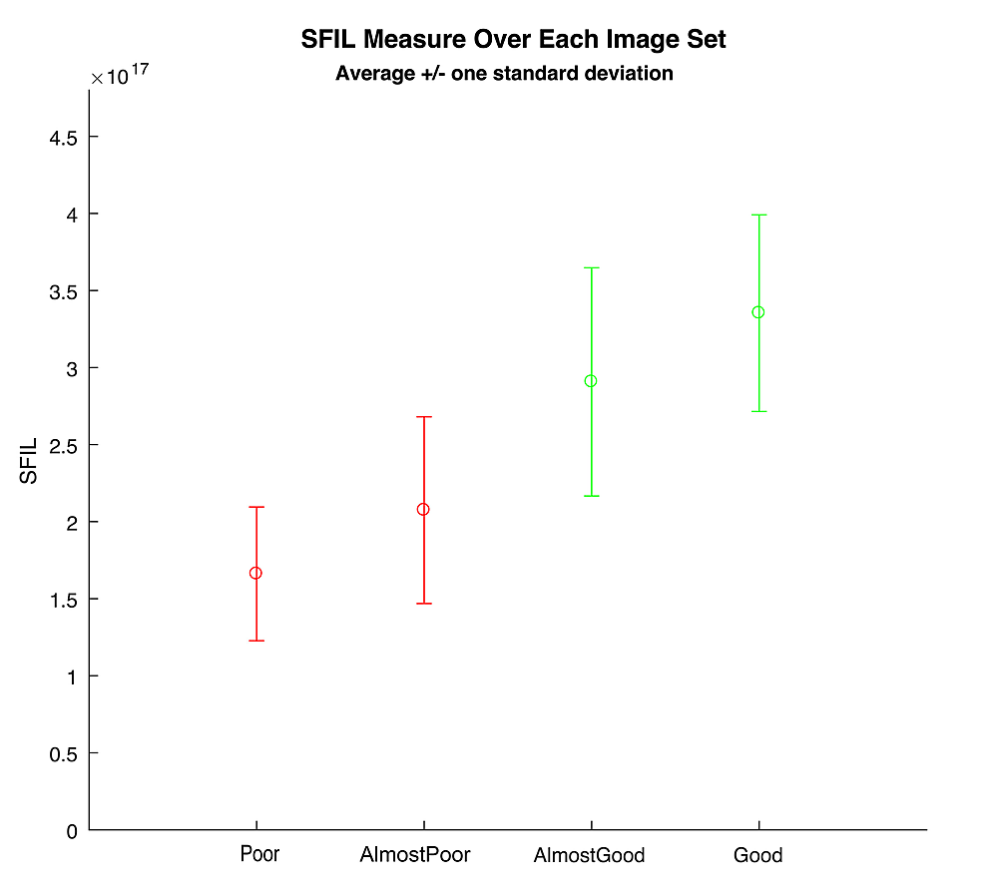
SFIL scoring of image sets, aligning with clinical classification. SFIL, steerable filters-based metrics.

A set of methods designed to estimate and score image focus in different ways were tested. These were drawn from published research on image focus. Focus measures can correlate well to a clear lumen that is being well visualized in front of the camera [9]. Testing these focus measures demonstrated that the "Poor" image contains very few crisp/in-focus visible edges while the "Good" image has crisp/in-focus edges visible [10]. We proceeded to test the various measures with focus ratings that most closely aligned with the clinical impressions. The focus measure, steerable filters-based metrics (SFIL), generated numerical scores for images aligned with the four categories, which were classified by the clinicians. Based on these results, we designed our algorithm to map SFIL results into four categories, with 1 = poor, 2 = almost poor, 3 = almost good, and 4 = good. At the end of this process, we had a program that could predict how a clinician would classify the images with 80-90% accuracy (Figure 2).

The next step was applying SFIL to video snippets. Video snippets that were 20-25 seconds in duration were scored with this algorithm. Using a computer program, these clips were extracted randomly from the original video files. Experienced endoscopists (C1 and C2) were blinded to their selection and required to score each snippet individually, as per the same four-point Likert scale. The algorithm was applied to produce a range of estimated per-frame scores across the duration of each clip. Scores generated by the clinicians and our algorithm were compared and analyzed.

Non-parametric testing was used with a Wilcoxon signed-rank test to evaluate inter-observer variability between the two clinicians. Goodman and Kruskal’s gamma was utilized to determine the association between each of the clinicians’ ratings and the SFIL result. Statistical analysis was performed using IBM SPSS Statistics for Windows, version 25.0 (IBM Corp., Armonk, NY).

## Results

A total of 500 video clips were extracted randomly from the available recorded videos. A total of 88 clips were excluded, as these were recordings outside the patient either before or after completion of the colonoscopy. Scores generated by C1, C2, and SFIL were compared. The algorithm provided a single distinct estimated classification score over the duration of the clip in 41% of all cases, with this matching the human classification 72% (C1) and 71% (C2) of the time. In 29% and 25% of all cases, respectively, our algorithm produced estimates being two-step ranges (e.g. "almost good to good") and three-step ranges (e.g. "poor to almost good"). In these cases, the returned ranges matched the human classifications 80-90% of the time. In 5% of all cases, we could not deduce any estimate.

Inter-clinician variability comparisons showed no discrepancy in 62.4% of cases, a slight discrepancy in 32.5% of cases (appropriated as a one band difference), and a significant difference in 5.1% of cases (>two band difference). The two independent clinician’s scores were within one band of each other 95% of the time. Statistical analysis using Wilcoxon signed-rank test showed no significant inter-clinician variability (Table 1).

**Table 1:** Comparison of inter-clinician variability in scores between clinician 1 (C1) and clinician 2 (C2). * Variation in the human classification of video clips between the two clinicians. 0^ = no difference in ranking; 1^ = single band difference; 2^ = two-band difference; 3^ = three-band difference.

Variability in inter-clinician scores of video clips				
		Frequency	Percent	
Degree of variation* in assessed clips between clinicians C1 and C2	0^	257	62.4	
	1^	134	32.5	
	2^	12	2.9	
	3^	9	2.2	

The comparison of SFIL with each clinician’s scores using a non-parametric model, i.e., Goodman and Kruskal’s gamma, revealed a significant correlation between C1 and SFIL (G = 0.644, p < 0.005) and a significant correlation between C2 and SFIL (G = 0.734, p < 0.005).

## Discussion

Colonoscopy has evolved to become the gold standard in the screening, diagnosis, treatment, and surveillance of colonic pathologies. The technique was first pioneered in 1969 by doctors William Wolff and Hiromi Shinya, with an electro-desiccative snare that facilitated polypectomies occurring soon after [11]. The technique proved revolutionary and experienced rapid spread internationally; more than 800,000 colonoscopies were performed in 2016-2017 in Australia alone [12].

The adoption of colonoscopy has been universal in the developed world. In contrast, developing countries rely more on opportunistic screening programs than the organized programs prevalent in developed countries [13]. Thai researchers demonstrated that despite an eight-fold increase in annual costs, colonoscopy was still likely to be more cost-effective than fecal immunochemical test (FIT)-focused screening. However, budgetary constraints and costs to citizens were two main factors that made a FIT-focused screening regime more likely to be adopted [14]. This example illustrates the challenge of prioritizing limited healthcare resources, highlighting the need for procedures like colonoscopies to be of high quality, meeting universally set objective performance standards.

Factors that influence the effectiveness of colonoscopies include thorough bowel reparation, careful inspection, and longer withdrawal times [15-17]. These factors influence the ADR [18], which determines intervals between subsequent colonoscopies with the potential to impact rates of interval cancers [19]. Cecal intubation, scope withdrawal time, and ADR are accepted parameters to objectively measure the quality of colonoscopies according to various Australian societies [12].

However, colonoscopies have several disadvantages. Lakoff et al. demonstrated that a negative colonoscopy is associated with an overall reduction in colorectal cancer (CRC), particularly in left-sided CRC for up to 14 years. Compared to this, right-sided CRC had a reduction of incidence of only seven years [20]. ADRs differ between specialties, gastroenterologists had higher polyp detection and removal rates than general surgeons [21]. This was noted in studies from the United States and correlated in the Australian context by Zorron Cheng Tao Pu et al. [22].

The reporting of quality measures is subjective and operator-dependent [23]. These studies and observations highlight the multitude of factors that can impact the effectiveness and quality of a colonoscopy. This has a direct bearing on the decision-making and clinical outcomes for patients. It impacts their management and translates to additional costs to the healthcare system.

Clear visualization of the mucosa is critical to performing a high-quality colonoscopy. The quality of the visualized image has been indirectly measured using various bowel preparation scoring scales. These scores reflect the ability to clearly see the lumen and colonic mucosa, which directly bears on the quality measures described above.

This study demonstrates a novel method to objectively report quality in colonoscopy, which aims to complement existing quality control methods. Objective measures of quality are important for several reasons. The first is allowing benchmarking across different practitioners, specialties, and institutions, thereby allowing a greater concordance in results. Current quality measures are inherently subjective and are thus poorly standardized, which makes result comparison between different institutions difficult. Introducing an objective measure of quality, therefore, allows for more efficient data sharing and would improve research endeavors between institutions.

The second is improving teaching by introducing an objective metric against which performance can be benchmarked and further refined. Khan et al. have written on the importance of feedback and debriefing on colonoscopy simulation training, whereby structured, objective feedback was directly correlated with clinical performance [24].

Finally, our study is preliminary and involves a limited number of videos. Greater numbers are needed for further validation. Therefore, we are hopeful that this pilot study can be used as a foundation for more research regarding focus measures in endoscopy and will pave the way for focus measures such as SFIL to be used alongside current colonoscopy quality measures. The method we describe needs refinement to reduce the margin for error and improve its accuracy. This would address one of the main limitations of the study. Another factor to consider is that different endoscopes or different generations of endoscopes and their attachments may have enough variation, which means more work is needed to standardize objective measures across other devices before they are safe for use.

## Conclusions

Colonoscopy is a potent tool used in colorectal cancer and adenoma screening plus surveillance that remains hampered by inter-observer variability. Software may therefore aid and augment luminal visualization improving factors such as ADR. While the method is still some steps away from rating colonoscopies or endoscopists, this study demonstrates that machine learning algorithms can objectively recognize the quality of luminal visualization during colonoscopy. We aim to apply this in the future as an aid to colonoscopy training and as a metric for quality measurement.
